# Evolution and development of cell walls in cereal grains

**DOI:** 10.3389/fpls.2014.00456

**Published:** 2014-09-11

**Authors:** Rachel A. Burton, Geoffrey B. Fincher

**Affiliations:** Australian Research Council Centre of Excellence in Plant Cell Walls – School of Agriculture, Food and Wine, University of AdelaideGlen Osmond, SA, Australia

**Keywords:** arabinoxylans, biosynthesis, cellulose, evolution, (1,3;1,4)-β-glucan, non-cellulosic polysaccharides

## Abstract

The composition of cell walls in cereal grains and other grass species differs markedly from walls in seeds of other plants. In the maternal tissues that surround the embryo and endosperm of the grain, walls contain higher levels of cellulose and in many cases are heavily lignified. This may be contrasted with walls of the endosperm, where the amount of cellulose is relatively low, and the walls are generally not lignified. The low cellulose and lignin contents are possible because the walls of the endosperm perform no load-bearing function in the mature grain and indeed the low levels of these relatively intractable wall components are necessary because they allow rapid degradation of the walls following germination of the grain. The major non-cellulosic components of endosperm walls are usually heteroxylans and (1,3;1,4)-β-glucans, with lower levels of xyloglucans, glucomannans, and pectic polysaccharides. Pectic polysaccharides and xyloglucans are the major non-cellulosic wall constituents in most dicot species, in which (1,3;1,4)-β-glucans are usually absent and heteroxylans are found at relatively low levels. Thus, the “core” non-cellulosic wall polysaccharides in grain of the cereals and other grasses are the heteroxylans and, more specifically, arabinoxylans. The (1,3;1,4)-β-glucans appear in the endosperm of some grass species but are essentially absent from others; they may constitute from zero to more than 45% of the cell walls of the endosperm, depending on the species. It is clear that in some cases these (1,3;1,4)-β-glucans function as a major store of metabolizable glucose in the grain. Cereal grains and their constituent cell wall polysaccharides are centrally important as a source of dietary fiber in human societies and breeders have started to select for high levels of non-cellulosic wall polysaccharides in grain. To meet end-user requirements, it is important that we understand cell wall biology in the grain both during development and following germination.

## INTRODUCTION

Two major differences distinguish the cell walls of cereal grains from those found in seeds of other higher plant species. Firstly, the cell walls of the Poaceae family, which includes the grasses as well as the economically important cereals, are fundamentally different in composition, compared with walls in dicotyledons and in most other monocotyledons. Secondly, walls in the grain of the Poaceae are usually quite different than those found in vegetative tissues. Here we will examine emerging evolutionary evidence and potential selection pressures that might account for these two levels of differences in wall composition in cereal grains.

Studies on the evolution and development of cell walls in cereal grains have been greatly accelerated through emerging technologies and genetic resources. In examining cell wall composition during grain development, it is clear that walls vary greatly in various parts of the grain and even between adjacent cells (Burton et al., 2010). It is therefore crucial to deploy new, high resolution *in situ* methods to define the heterogeneity of wall composition in plant material that contains different cell types. Thus, sophisticated methods for determining polysaccharides present in walls during grain development are under development. For example, there has been a recent surge in the availability of reliable antibodies and carbohydrate binding modules that detect specific epitopes on wall polysaccharides (Verhertbruggen et al., 2009; Pattathil et al., 2010) and can therefore be used to distinguish different wall compositions in immunocytochemical labeling at both the light and electron microscopy levels (Wilson et al., 2012). In addition, there are new imaging methods with improved resolution, such as Fourier-transform infra-red (FT-IR), Raman and nuclear magnetic resonance (NMR) spectroscopy, and matrix-assisted laser desorption/ionization mass spectrometry imaging (MALDI–MSI). The use of these spectroscopic and immunocytochemical methods have confirmed that there is no such thing as a “standard” homogeneous cell wall in any tissue and this is no less true in the various cell types of cereal grains.

Evolutionary studies on cell wall polysaccharides have been greatly assisted by the identification of genes that encode polysaccharide synthases that are responsible for wall synthesis (Pear et al., 1996; Dhugga et al., 2004; Burton et al., 2006; Sterling et al., 2006; Doblin et al., 2009) and the recognition that the synthases are encoded by families of genes (Richmond and Somerville, 2000; Hazen et al., 2002). Our knowledge of the genes that mediate wall polysaccharide biosynthesis is increasingly assisted by the availability of genome sequences of important cereal and grass species, high throughput transcript profiling, and by the availability of rapidly expanding genetic resources for cereal species, including mutant libraries. Further exploration of non-crop grass species and the increasing use of grain development mutants, coupled with the emerging imaging and transcript analysis capabilities, will surely throw up more surprises and help us unravel the complex process of grain development. Here, we briefly review the current knowledge of wall composition in cereal grain and consider the evolutionary origins of diverse grain compositions.

## MORPHOLOGY OF WALLS IN THE GRAIN

Large variations are observed in cell wall compositions between different species of grasses. Until recently most attention was focused on walls of the cereals, including wheat (Mares and Stone, 1973), barley (Fincher, 1975), and rice (Shibuya and Iwasaki, 1985). More recently, information has been published on endosperm walls from the grass *Brachypodium distachyon* (Guillon et al., 2011). Significant differences are observed in the polysaccharide compositions of the walls in these species and in the morphology of the endosperm although only a relatively narrow range of forms have been described. Indeed, Terrell (1971) surveyed 169 grass genera and found that a significant proportion of these had persistent liquid, soft, or semi-solid endosperm, the investigation of which surely has implications for grain quality and for the field of cell wall biology in general. The values in **Table 1** illustrate the differences in wall compositions between grains of selected grass species and between vegetative tissues and fruit of grass and dicotyledonous species.

**Table 1:** Selected comparisons of polysaccharide compositions in walls of vegetative tissues, fruit, and grains/seeds (% w/w).

Tissue	Hetero-xylan	(1,3;1,4)-β-Glucan	Cellulose	Hetero-mannan	Pectin	Xyloglucan	Reference
Barley coleoptiles (4 days)	32	10	35	nr	12	10	Gibeaut et al. (2005)
Barley aleurone	71	26	2	2	nd	nd	Bacic and Stone (1981)
Barley starchy endosperm	20	70	3	2	nd	nd	Fincher (1975)
Maize internodes	46	3	35	2	trace	6	Zhang et al. (2014)
*Brachypodium* whole grain	4.7	42.4	6	trace	nr	nr	Guillon et al. (2011)
Rice endosperm	32	nr	36.3	nr	7.3	nr	Shibuya and Iwasaki (1978)
*Arabidopsis* leaves	4	0	14	nr	42	20	Zablackis et al. (1995)
Grape berries (114 DPA)	7	0	31	3	45	8	Nunan et al. (1998)

*nd, not detected; nr, not reported.*

**FIGURE 1:**
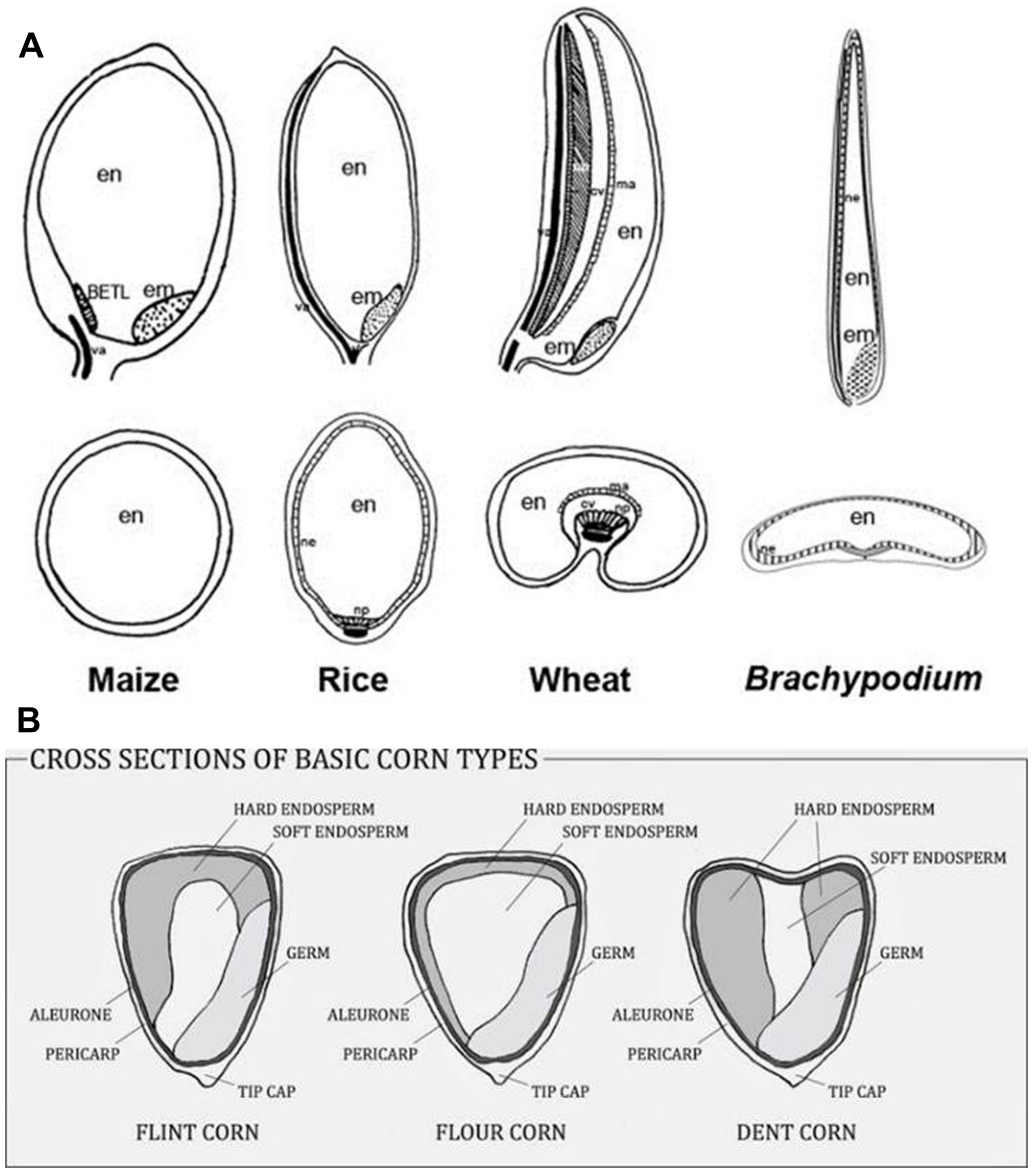
**(A)** Examples of different grain morphologies. **(B)** Hard and soft endosperm proportions vary in maize kernels. Reproduced with permission from Hands and Drea (2012) and http://www.deductiveseasoning.com/2014/03/planting-and-growing-corn-for-nutrition.html. In panel **(A)**, em, embryo; en, endosperm; ma, modified aleurone; cav, cavity; va, vasculature; ne, nucellar epidermis; np, nucellar projection; BETL, basal endosperm transfer layer.

The starchy endosperms of most economically important cereals display a range of morphological forms (**Figure 1A**) and a range of cell shapes and sizes across the grain. In barley there are wings of irregularly shaped starchy endosperm cells that flank a central core of prismatic cells overlying the transfer cells (TC; Becraft and Asuncion-Crabb, 2000). The outer endosperm cells in wheat are prismatic whilst the inner cells are rounded (Toole et al., 2007). In rice grain the endosperm cells are radially symmetrical and so appear to be tube-like (Srinivas, 1975). In sorghum, hard or translucent endosperm tissue surrounds a softer, opaque core (Waniska, 2000). In the former there are no air spaces and the starch granules are packed in tightly. In the softer core region there are large intergranular air spaces that affect both the properties of the tissue and the way that it reflects light. Maize kernels possess the same features (**Figure 1B**) and sorghum and maize grain can be dominated by one particular type of endosperm and thus can be predominantly soft or hard (Evers and Millar, 2002). In the same way, barley varieties can be described as mealy or steely (Ferrari et al., 2010). Grain hardness and strength, for example in sorghum and maize, is related to the packing of the starch granules within their protein matrix, rather than to the cell walls (Chandrashekar and Mazhar, 1999).

## WALL COMPOSITION IN GRAIN DIFFERS FROM THAT IN VEGETATIVE TISSUES

In most dividing cells of vegetative tissues of higher plants, a callosic cell plate forms between the newly separated nuclei (Waterkeyn, 1967; Morrison and O’Brien, 1976). The cell plate acts as a scaffold on which the new wall is built. Cellulosic and non-cellulosic polysaccharides are deposited on both sides of the cell plate until the nascent wall eventually separates the daughter cells. The cell plate is compressed to a thin middle lamella layer that lies between walls of the two daughter cells. Wall deposition continues as the cells expand, but at this stage the wall remains relatively thin to allow this expansion to occur and is usually referred to as the primary wall. As cell expansion ceases, wall deposition continues in many cells to form a much thicker and stronger secondary wall, which can be further strengthened by the deposition of lignin and through lamination of parallel sheets of cellulose microfibrils that are oriented in different directions.

As noted above, the first distinguishing feature of walls in grasses compared with other plant species is related to their composition. Although pectic polysaccharides are amongst the earliest wall components to be deposited in both dicotyledons and monocotyledons, the levels of pectic polysaccharides in the walls of grasses decline during wall development to low levels relative to those observed in dicot walls. Other non-cellulosic polysaccharides are deposited during primary wall formation, including xyloglucans, heteromannans, and heteroxylans. In primary walls of the grasses, pectic polysaccharides and xyloglucans are found at relatively low levels, while the heteroxylans appear to form the core non-cellulosic polysaccharides of most walls (Burton and Fincher, 2009). An additional wall polysaccharide is often deposited, namely the (1,3;1,4)-β-glucans. This polysaccharide is not widely distributed outside the Poaceae and the genes that mediate its biosynthesis are believed to have evolved relatively recently. The wall composition of the grasses can be contrasted with the walls of *Arabidopsis*, where xyloglucans appear to be the core non-cellulosic polysaccharides, pectic polysaccharides remain relatively high, and the levels of heteroxylans are low (Zablackis et al., 1995).

**FIGURE 2:**
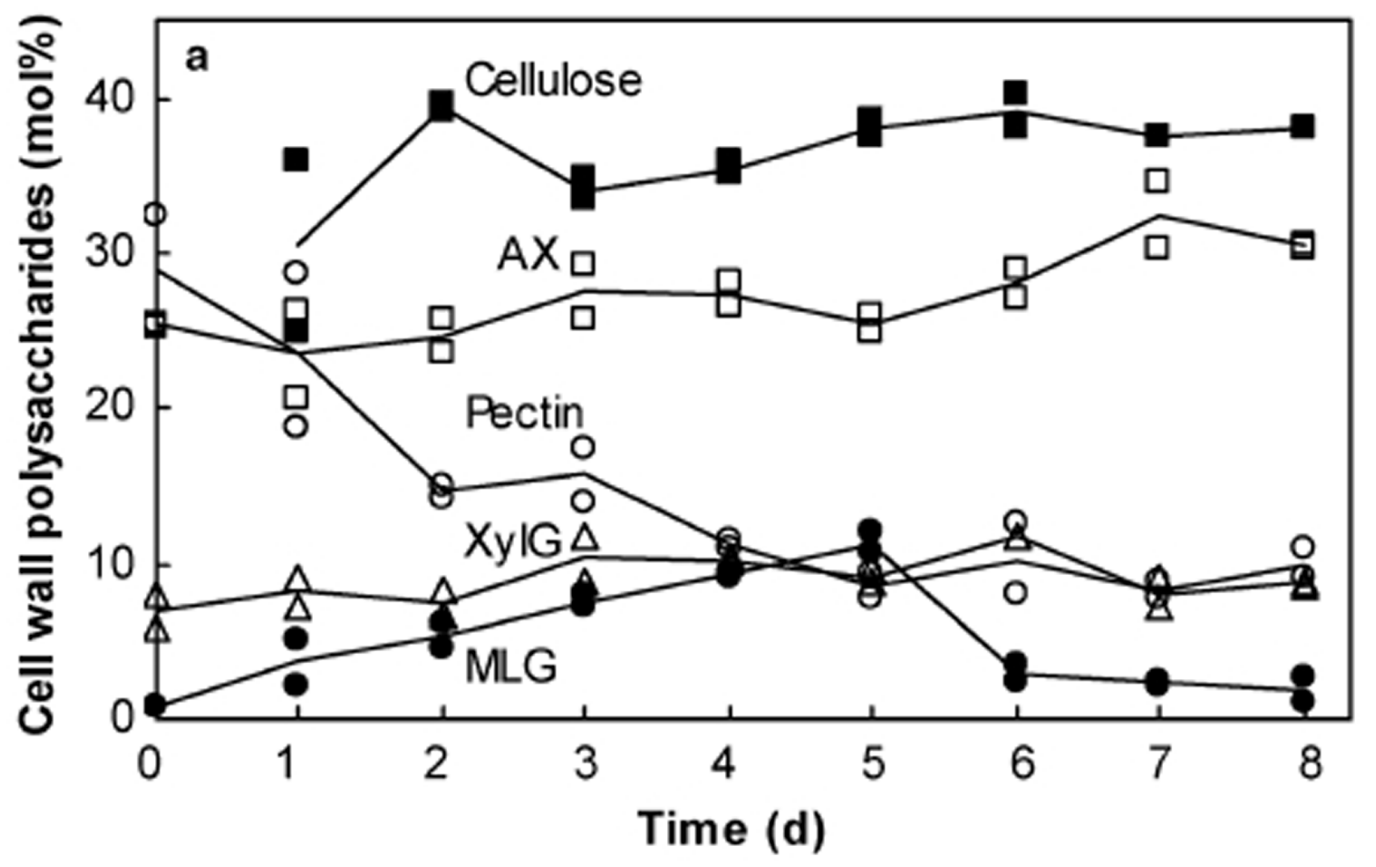
**Changes in cell wall composition during the development of barley coleoptiles.** Compositions were deduced from data obtained by alditol acetate, methylation and acetic–nitric acid analyses. Changes in cellulose (filled square), arabinoxylan (AX, open square), pectic polysaccharides (pectin, open circle), xyloglucan (XylG, triangle), and (1,3;1,4)-β-glucan (MLG, filled circle) are shown. Reproduced with permission from Gibeaut et al. (2005).

The differences are exemplified in developing coleoptiles of barley (Gibeaut et al., 2005), where pectic polysaccharides decrease from about 30% w/w to just a few percent of walls over 6 days. Heteroxylan levels remain at about 30% w/w throughout coleoptile development, while xyloglucan levels are generally 10% w/w or less (**Figure 2**; Gibeaut et al., 2005). Similar results were reported for the composition of walls in elongating maize internodes, which can also be viewed as a useful system for monitoring developmental changes in wall composition in vegetative tissues of the Poaceae (Zhang et al., 2014).

**FIGURE 3:**
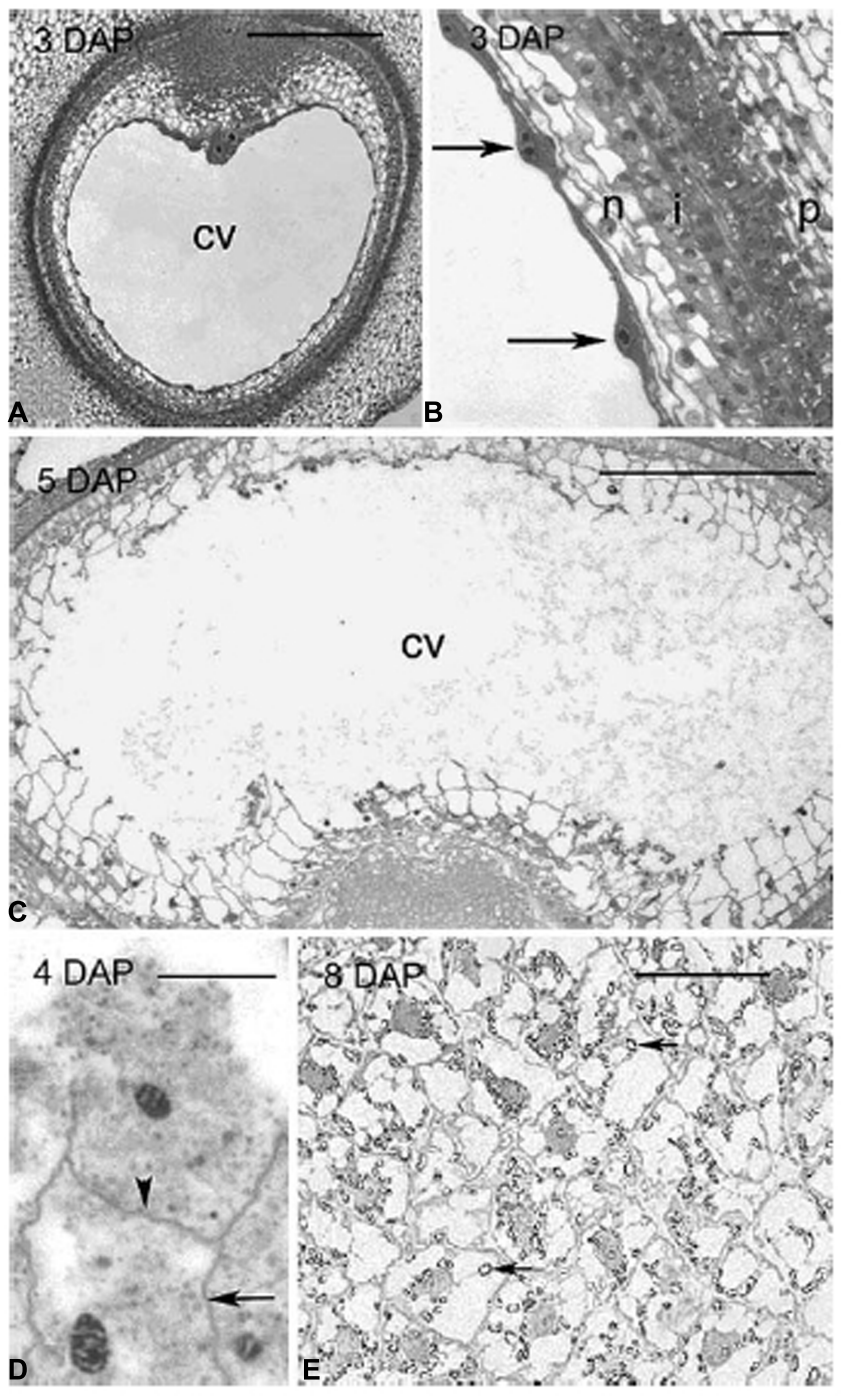
**Different stages of endosperm development in barley.** Light micrographs of sections through barley grains showing stages of endosperm development from 3 to 8 DAP. **(A)** 3 DAP: a thin layer of syncytial cytoplasm surrounds a large central vacuole. **(B)** Details of the syncytium in **(A)**. Arrows indicate the position of nuclei along the perimeter of the central cell, all enclosed within discrete layers of maternal tissue. **(C)** 5 DAP: cellularization occurs centripetally with repeated cycles of anticlinal wall formation, mitosis and periclinal wall formation.** (D)** 4 DAP: shows the wavy appearance of anticlinal walls (arrow) and a periclinal wall (arrowhead) separating two recently divided daughter nuclei. **(E)** 8 DAP: the endosperm was fully cellularized and starch granules (arrows) had accumulated within each cell. cv, central vacuole; i, integuments; n, nucellus; p, pericarp. Scale bars = 300 μm **(A,C)**, 50 μm **(B,E)**, 20 μm **(D)**. Reproduced with permission from Wilson et al. (2006).

The second distinguishing feature of wall composition in the Poaceae is seen in comparisons between vegetative tissues and grain and, more particularly, the starchy endosperm. Botanically, grains are one-seeded fruits, or caryopses (Esau, 1977). Formation of cell walls in the developing endosperm proceeds via a completely different developmental program from other tissues. Fusion of a sperm cell with two haploid central cell nuclei gives rise to a triploid endosperm nucleus. Repeated nuclear division produces many nuclei in a syncytium, which is essentially a cavity in the caryopsis. In most cases, cellularization follows, where callosic cell walls are laid down from the outside in, simultaneously separating the nuclei and apportioning them evenly into cells until the newly formed endosperm walls eventually meet at a central point to fill the coenocyte, as exemplified by rice (Brown et al., 1994), sorghum (Paulson, 1969), and barley (Wilson et al., 2006; **Figure 3**). In both cellularizing barley and rice endosperm callose is believed to be the major component of the cell walls that grow around the nuclei in the syncytium. In barley callose is found along the central cell wall at 3 days after pollination (DAP); it is present in the first and subsequent anticlinal walls from 4 DAP, in the periclinal walls at 5 DAP and disappears at 6 DAP, except in the vicinity of plasmodesmata (Wilson et al., 2006).

Callose often re-appears much later during barley and wheat grain development (Fulcher et al., 1977; Bacic and Stone, 1981). At 28 DAP, newly deposited patches of callose are detected at irregular spacings along the aleurone–subaleurone interface of barley grain (Wilson et al., 2012). The function of these deposits is unclear but they may represent a wound response to the osmotic stresses imposed by desiccation of the maturing grain or by periods of water stress during grain maturation (Fincher, 1989).

Despite the different cellular developmental patterns in the grain, the walls of the mature grain are still composed of the polysaccharides observed in vegetative walls. However, in the endosperm of many grass species, the amount of cellulose is reduced to just a few percent on a weight basis, which can be contrasted with cellulose contents of 30% (w/w) or more of primary walls in vegetative tissues (Fincher, 2009). The low cellulose content in the endosperm is consistent with the fact that these cells have no load-bearing function, as distinct from walls in barley coleoptiles or maize stalk internodes, and because it is important for walls of endosperm cells to be quickly degraded in the germinated grain. High levels of cellulose in these walls would almost certainly slow their rate of degradation following germination. However, it must be noted that walls in the starchy endosperm of grain do have to withstand pressures exerted by grain expansion and later by dehydration as the grain matures, and such stresses may trigger changes in the matrix polysaccharides of the wall.

## WALL COMPOSITION IN DIFFERENT TISSUES OF THE GRAIN

Most of the discussion above has been focused on the development of cell walls in the starchy endosperm of grains of the Poaceae. However, as the grain develops several other specific cell types can be distinguished. These include, in addition to the starchy endosperm cells, which are the main repository for starch and storage protein, TC, which are clustered around the vascular network that feeds the growing grain, aleurone cells, which envelop the starchy endosperm and are rich in oil and protein bodies, sub-aleurone cells that arise through periclinal division of the aleurone cells, and finally the embryo itself, which is comprised of many organ-specific vegetative tissues. Information on the cell walls of these tissues is not extensive, but some interesting data are emerging.

### ALEURONE LAYER

Aleurone cells form a layer around the starchy endosperm that varies from one to three or four cells in thickness, depending on the species, and are indeed components of the endosperm as a whole. Aleurone cells are typically cuboid in shape with much thicker cell walls, usually at least twice the thickness of those in the central starchy endosperm. Aleurone cells contain a dense granular cytoplasm comprised of aleurone grains and small vacuoles containing inclusion bodies (Olsen, 2004). They are rich in proteins and oil but contain no starch and, unlike the cells of the starchy endosperm which undergo programmed cell death (Young and Gallie, 2000), they remain living in the mature grain. This is essential if they are to perform their key role in grain germination, where they synthesize and release a range of hydrolyzing enzymes that are responsible for mobilizing the storage polymers of the starchy endosperm. Aleurone cells usually remain triploid, unlike the starchy endosperm cells, which undergo endoreduplication and become polyploidy in nature (Olsen, 2001).

The walls of aleurone cells in mature barley and wheat grain have two quite distinct layers (Taiz and Jones, 1973; Bacic and Stone, 1981). The inner layer is thinner and may have higher concentrations of (1,3;1,4)-β-glucans (Wood et al., 1983). The thicker outer layer of the aleurone wall may be enriched in arabinoxylans, although ferulic acid residues were believed to be evenly distributed across the two wall layers (Fincher, 1989). The two layered structure of aleurone walls might be important during grain germination, when the thick outer layer is rapidly dissolved, while the thin, inner layer remains intact. The outer layer might be removed to facilitate the secretion of newly synthesized hydrolytic enzymes into the starchy endosperm (Van der Eb and Nieuwdorp, 1967; Gubler et al., 1987), while the retention of the thin inner layer might be necessary to maintain the physical integrity of the aleurone cells until their role in enzyme secretion is complete (Fincher, 1989). Walls of the scutellar epithelium layer, which is important in the secretion of hydrolytic enzymes into the starchy endosperm early after germination (McFadden et al., 1988), have morphological features that are similar to those of the aleurone and it is likely that the walls of the scutellar epithelium have a similar composition to those of the aleurone layer (Fincher, 1989).

The developmental cues for aleurone cells are complex and not yet fully understood. In wheat, they have a specific molecular signature by 6 days post anthesis, conferred by their position in the “surface layer” (Gillies et al., 2012). However, aleurone cell fate remains plastic up to the last cell division and specific signals are necessary to maintain cell identity (Becraft and Yi, 2011). In barley grain, aleurone cells are present at 10 DAP and their walls continue to thicken until 22 DAP when grain maturation begins (Wilson et al., 2012). Many cereals also have a zone of cells that separate the true aleurone from the starchy endosperm cells. These subaleurone layers arise from periclinal division of the aleurone cells (Becraft and Asuncion-Crabb, 2000) and in barley they are present by 14 DAP (Wilson et al., 2012). Sub-aleurone cells are larger than aleurone cells but smaller than starchy endosperm cells, and contain small starch granules and protein bodies.

The developmental signals that dictate the number of cell layers and hence the thickness of the aleurone layer overall are gradually being unraveled (Sabelli and Larkins, 2009). Aleurone layer thickness, the number of cell layers therein and the regularity of thickness has been examined in a range of cereals by Hands et al. (2012). Barley was found to be the only grain to consistently possess a layer more than one cell in thickness. The non-cultivated species *B. distachyon* and *Festuca pratensis* have markedly more disorganized and irregular aleurone layers, which may imply that there is a correlation between regularity of shape and domestication, since this trait may have been selected to meet certain grain quality parameters, such as speed of germination and endosperm mobilization (Hands et al., 2012). However, our knowledge of grain ultrastructure in non-crop species of the Poaceae is generally poor but increasing the number of cell layers in aleurone layers could be beneficial. Approximately half the volume of cereal bran is comprised of aleurone tissue and since this is the most dietary beneficial part of the bran, rich in proteins, oils, and other phytonutrients, increasing the amount further is desirable in human health and animal nutrition (Okarter and Liu, 2010). However, there are also milling considerations, since aleurone cell walls are so thick the cells may remain intact and their contents unobtainable (Minifie and Stone, 1988).

The core polysaccharides found in aleurone cell walls are also arabinoxylans, although relatively high levels of (1,3;1,4)-β-glucan are found in wheat and barley grain. Early work in which aleurone cells were isolated and analyzed showed that aleurone walls from wheat and barley contained about 65% arabinoxylan and about 28% (1,3;1,4)-β-glucan; cellulose and glucomannan levels were again very low (Bacic and Stone, 1981). Several groups have used immunolabeling, Raman spectroscopy, and IR microspectroscopy to monitor changes in aleurone call walls, *in situ*, during the development of wheat grain. Aleurone walls are more heterogeneous early in grain development compared with those at maturity (Jamme et al., 2008). Antibody labeling indicated the presence of the pectic polysaccharide epitopes RGI, (1,5)-α-arabinan and (1,4,)-β-galactan in the aleurone, particularly on the inner surface of the cell wall, and in the pericarp in mature grain (Jamme et al., 2008; Chateigner-Boutin et al., 2014).

Strong autofluorescence has long been known in aleurone and is attributable to high levels of the phenolic acids, ferulic acid, and *p*-coumaric acid in mature aleurone walls in wheat (Fulcher et al., 1972; Bacic and Stone, 1981; Robert et al., 2011) and other cereals. These phenolic compounds have been examined more closely by Jaaskelainen et al. (2013) using *in situ* optical and Raman microscopy. In the aleurone cells of both barley and wheat, the anticlinal walls contain high amounts of phenolic acids compounds, with much less in the inner periclinal walls. In barley, phenolic compounds were particularly strong in the outer periclinal walls. Ferulic acid, and indeed arabinoxylan, were first detected in the newly differentiated aleurone walls in barley grain at 12 DAP (Wilson et al., 2012). Jaaskelainen et al. (2013) confirmed that there is no (1,3;1,4)-β-glucan in the middle lamella of aleurone walls but that arabinoxylan is enriched here and in the outer cell wall layers.

### TRANSFER CELLS

**FIGURE 4:**
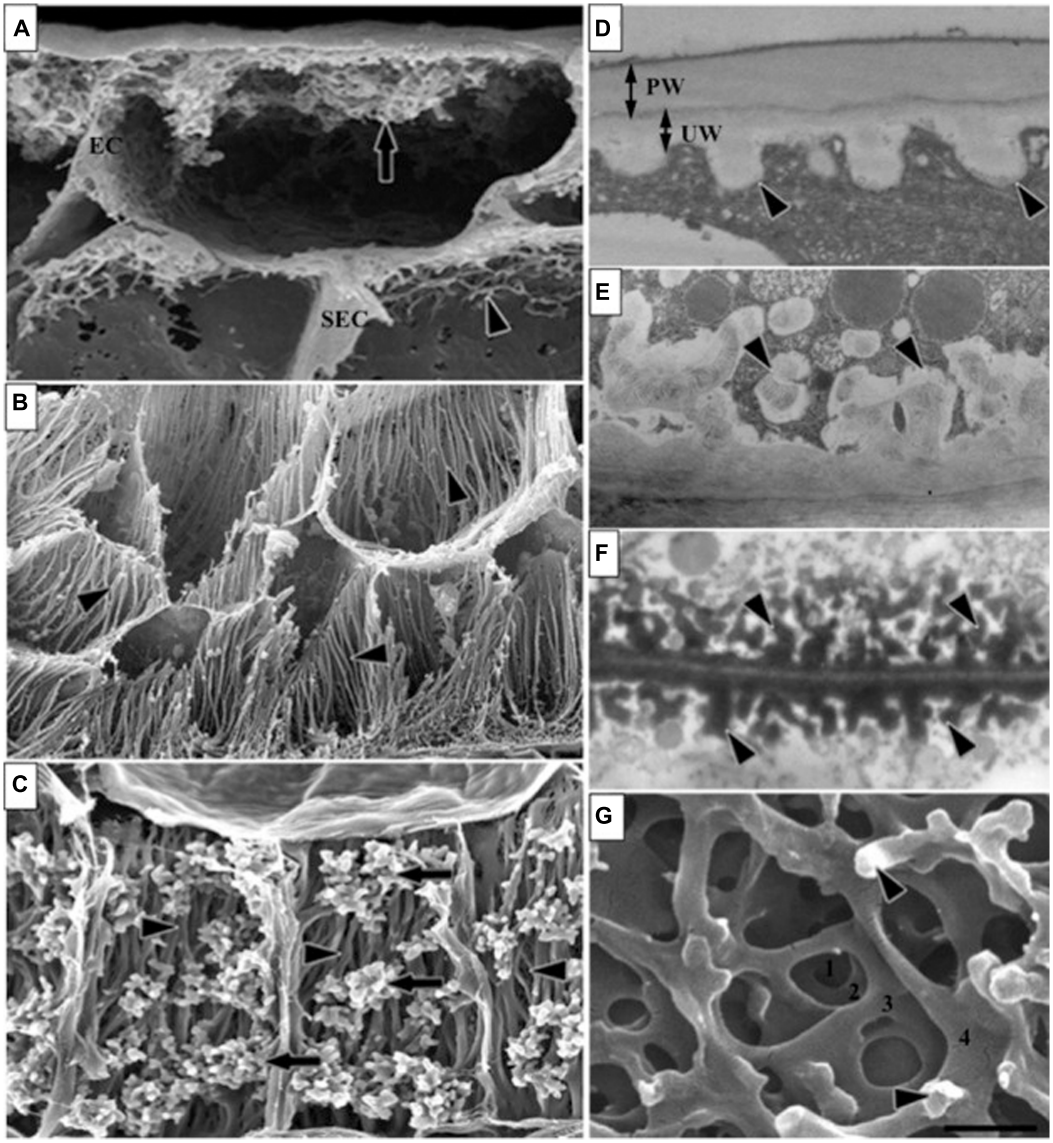
**Different types of transfer cells (TC) in cereals and other seeds.** These images of TC of developing seeds illustrate various ingrowth wall morphologies. **(A)** Epidermal transfer cells (ETC) of a *Vicia faba* cotyledon with an extensive reticulate ingrowth wall labyrinth including clumps of ingrowth material (arrow) and smaller wall ingrowths in the subepidermal cells (SEC; arrowhead). **(B)** Basal endosperm TC of *Zea mays* exhibiting flange wall ingrowth morphology; arrowheads indicate small lateral protrusions from the linear ribs (modified after Talbot et al., 2002). **(C)** Thin-walled parenchyma TC located at the inner surface of the inner seed coat of *Gossypium hirsutum* with wall ingrowth flanges (darts) extending the length of each cell on which are deposited groups of reticulate wall ingrowths (arrows; modified after Pugh et al., 2010). **(D–F)** Transmission electron microscope images of portions of transverse sections of TC: **(D)** the outer periclinal wall of an adaxial epidermal cell of a *V. faba* cotyledon induced to trans-differentiate to a transfer cell morphology displaying primary wall (PW) and uniform walls (UW). **(E)** Small papillate ingrowths (darts) of a seed coat transfer cell of *V. faba* exhibiting reticulate architecture. **(F)** Antler-shaped reticulate wall ingrowths (darts) of a nucellar projection transfer cell of a developing *Triticum turgidum* var. durum seed (modified after Wang et al., 1994). **(G)** Field emission scanning electron microscope image of the cytoplasmic face of the reticulate ingrowth wall labyrinth of an abaxial epidermal transfer cell of a *V. faba* cotyledon following removal of the cytoplasm and dry cleaving (for method see Talbot et al. (2001), image modified after Talbot et al. (2001)) where the darts indicate ingrowth papillae on the most recently deposited wall layer. Single scale bar for **(A,B)** = 2.5 μm; for **(C)** = 5 μm; for **(D,E)** = 1 μm; for **(F)** = 0.25 μm; for **(G)** = 0.5 μm. Figure legend and images reproduced with permission from Andriunas et al. (2013).

Transfer cells provide the major route for nutrient acquisition by the developing endosperm and they are therefore a key determinant of grain filling. TCs are present in a range of tissues in many plant species and they can be classified into two types, namely reticulate and flange-like. Through the deposition of secondary cell wall material, both types develop a massively expanded surface area to facilitate the transfer of nutrients. Wang et al. (1994) estimated that the plasma membrane surface area increases up to 22-fold. Reticulate types are exemplified by TCs found in *Vicia faba* cotyledons whereas flange-like types are typically found in cereals (McCurdy et al., 2008; **Figure 4**). Reticulate TCs arise from re-differentiation of epidermal cells (Oﬄer et al., 1997), which is a very different pathway from the direct differentiation of flange-like TCs from endosperm cells in developing cereal grains. The latter occurs opposite the nucellar projection as early as 5 DAP in barley, when the first wall ingrowths appear in the syncytium (Thiel et al., 2012b). By 7 DAP the TC walls are enlarged with net-like and branched strands on the inner wall and TCs represent 6.7% of the total endosperm volume; they increase in area ninefold between 5 and 10 DAP. By 10 DAP the walls are thicker with rib-shaped projections and cells are flattened in parallel with the long axis of the grain; by 12 DAP the wall thickenings are asymmetric and irregularly spaced and the flanges have fused; and by 14 DAP TCs represent a much lower proportion of the total endosperm volume at just 0.9% (Thiel et al., 2012b). Wheat TCs develop in a similar fashion to those in barley (Zheng and Wang, 2011), whilst maize TCs present a dense network of flanges and are found in the basal endosperm (Zheng and Wang, 2010), and rice TCs are found in the aleurone layers in the dorsal region of the grain adjacent to the major vascular bundle in the pericarp. Development of TCs in rice is uneven but they also show wall in-growths (Hoshikawa and Wang, 1990).

The deposition of layers of material onto the original wall in TCs has been defined as secondary wall thickening. This occurs widely in many vegetative parts of the plant as cell expansion ceases and wall deposition continues to form a much thicker and stronger secondary wall, which can be further strengthened through the deposition of lignin and *via* lamination. We know that the major polysaccharides laid down through secondary thickening are cellulose and heteroxylans, with the deposition of lignins to further strengthen and, in some cases, to waterproof the wall. Although we know much less about the composition of TC walls, it would seem likely that they do not resemble a typical secondary wall. Significantly, lignin is absent and in wheat, arabinoxylan is the predominant component from 5 to 23 DAP (Robert et al., 2011), and is more highly substituted than the arabinoxylan in the walls of the aleurone layer. After 23 DAP, the TC walls become enriched in (1,3;1,4)-β-glucan, which also occurs in the aleurone, and again this is not typical of secondary cell walls in other parts of the plant.

Recently, laser-microdissection methods have been used successfully to define tissue-specific transcripts and allow metabolite profiling of TCs in barley (Thiel et al., 2012a; Thiel, 2014).

## MINOR WALL POLYSACCHARIDES IN THE GRAIN

The core non-cellulosic wall polysaccharides of the grain are the heteroxylans and, in some cases, (1,3;1,4)-β-glucans, while cellulose contents are usually low, as noted above (Fincher and Stone, 2004). However, there is one notable variant when it comes to wall composition in the starchy endosperm of grasses. The endosperm walls of mature rice grain are comprised of significant amounts of cellulose, up to 30% as reported by Shibuya and Nakane (1984). Cellulose is also present at higher levels during the very early stages of barley grain development (Wilson et al., 2006).

Although arabinoxylan and (1,3;1,4)-β-glucan predominate in cereal grain cell walls, we are starting to discover the presence of other polysaccharides which, although only minor components of the walls, may represent key determinants of wall plasticity and other properties. Thus, levels of pectic polysaccharides, heteromannans, and xyloglucans are low in many grains, including wheat and barley (Mares and Stone, 1973; Fincher, 1975). Again an exception here appears to be rice, which contains relatively high levels of pectin (Shibuya and Nakane, 1984) and xyloglucan (Shibuya and Misaki, 1978). Xyloglucan can also be detected in barley grain during early grain development, but appears to be transitory in nature. It first appears at 3 DAP in the central cell wall but is undetectable by 6 DAP (Wilson et al., 2012). Mannans first appear in barley endosperm walls at 5–6 DAP, after cellularization is complete and, based on the accumulation of mannose, mannans, or glucomannans continue to be deposited at low levels up to 20 DAP (Wilson et al., 2012); the final levels of mannans or glucomannans in mature wheat and barley grain are about 2–3% w/w (Mares and Stone, 1973; Fincher, 1975).

Small but significant pectic deposits have recently been reported in wheat grain (Chateigner-Boutin et al., 2014). Pectins have previously been reported in rice endosperm cell walls (Shibuya and Nakane, 1984) and in *B. distachyon* (Guillon et al., 2011) but little is known about their presence or otherwise in the majority of cereal grains. Pectins are complex, multi-domain polysaccharides that bear many different epitopes (Caffall and Mohnen, 2009). Chateigner-Boutin et al. (2014) used antibodies that recognize specific pectic epitopes on sections of developing and mature wheat grains. The inclusion of pre-labeling enzymatic digests with lichenase and xylanase to remove a portion of the major polysaccharides (1,3;1,4)-β-glucan and arabinoxylan proved to be a key step in rendering the pectic epitopes accessible. In the developing grain LM20, which recognizes methyl-esterified homogalacturonan (HG; Verhertbruggen et al., 2009), labeled the pericarp and early endosperm walls, where elasticity would be required. In older grain, large bodies containing unesterified HG, as detected by LM19, were found located in the subcuticle layer, and the reason for their presence here is currently unclear (Chateigner-Boutin et al., 2014).

## EVOLUTIONARY DIFFERENCES IN HETEROXYLANS IN THE GRAIN

Consistent with the low cellulose content of endosperm walls, the levels of the core wall polysaccharide in the Poaceae, the heteroxylans, are relatively higher in the starchy endosperm, while the levels of the core polysaccharides of dicotyledonous plants, pectic polysaccharides, and xyloglucans, are generally much lower. Indeed, heteroxylans are found in all walls of the grasses and are the major non-cellulosic polysaccharide in most walls. However, there is evidence of evolutionary forces at work on the heteroxylans of the Poaceae. In dicotyledonous plants, glucuronoarabinoxylans are abundant and in some cases glucuronyl residues predominate. In the grasses, two types of heteroxylans can be distinguished. Glucuronoarabinoxylans are relatively abundant in the outer, pericarp-testa layers of the grain and in bran, while arabinoxylans are the major non-cellulosic polysaccharides of the aleurone and starchy endosperm cell walls (Fincher and Stone, 2004).

The species best characterized for arabinoxylan is wheat, where isolated endosperm walls comprise about 70% of this polysaccharide (Mares and Stone, 1973). The (1,4)-β-xylan backbone of the polysaccharide displays both structural and spatial heterogeneity with regard to its degree of substitution and this heterogeneity varies throughout endosperm development, as assessed by enzyme mapping, FT-IR, and Raman microscopy and NMR spectroscopy (Toole et al., 2007, 2009, 2010, 2012). Early in endosperm development more of the backbone (1,4)-β-linked xylosyl residues are di-substituted with arabinofuranosyl residues at the *O-2* and *O-3* positions, but as the grain matures, a higher degree of mono-substitution at the *O-3* position is observed, possibly to allow more inter-chain interactions to occur to withstand mechanical stresses as the grain dries out. Ferulic acid and to a lesser extent *p*-coumaric acid residues are ester-linked at *O-5* of some of the *O-3* mono-substituted arabinosyl groups and it has been reported that these can form covalent cross-links between arabinoxylan chains through oxidative dimerization (Iiyama et al., 1990). There is a gradient of arabinoxylan substitution patterns across the grain as prismatic cells give way to round cells (Toole et al., 2010). Barley endosperm cell walls also contain about 20% arabinoxylan (Fincher, 1975) and show subtle inter-species variation in the types and amounts of backbone substitutions (Izydorczyk, 2014). This is also evident in rye grain, which has a much higher ratio of mono- to di-substitutions than wheat (Rantanen et al., 2007).

The substitution of the extended (1,4)-β-xylan backbone with arabinofuranosyl residues sterically hinders the aggregation of the (1,4)-β-xylan chains into insoluble microfibrils and results in the formation of a long, asymmetrical polysaccharide that is partly soluble in water and can form gel-like structures in the cell wall matrix (Fincher and Stone, 2004). As expected, the degree of substitution of the (1,4)-β-xylan backbone will affect the physical properties of the polysaccharide and, in particular, its solubility. Highly substituted, soluble arabinoxylans, which have a characteristically high arabinose:xylose ratio, are found in the endosperm cells of the grain, while arabinoxylans with lower degrees of substitution are less soluble and are located in the outer layers of the grain (Fincher and Stone, 2004; Izydorczyk, 2014).

## EVOLUTION OF (1,3;1,4)-β-GLUCANS IN THE GRASSES

Another key difference in walls of cereal grains compared with other seeds is the presence of (1,3;1,4)-β-glucan. This polysaccharide has an interesting distribution in the plant kingdom (Harris and Fincher, 2009). It is found in many species of the Poaceae but is also occasionally found in other Poales, and in lower plants such as the *Equisetum* spp. horsetail ferns (Trethewey et al., 2005; Fry et al., 2008; Sørensen et al., 2008), bryophytes (Popper and Fry, 2003), some fungi (Pettolino et al., 2009), brown, green and red algae (Lechat et al., 2000; Eder et al., 2008; Popper and Tuohy, 2010), and lichens (Stone and Clarke, 1992). This distribution pattern of (1,3;1,4)-β-glucans in higher and lower plants is suggestive of convergent evolution. The (1,3;1,4)-β-glucans seem to have been widely adopted only in the Poaceae, where one might conclude there is positive selection pressure to retain the polysaccharide in the walls.

The (1,3;1,4)-β-glucans of the grasses are comprised of an unsubstituted chain of glucosyl residues linked either through (1,4)-β- or (1,3)-β-linkages. About 90% of the polysaccharide chain is comprised of cellotriosyl (DP3) and cellotetraosyl (DP4) units that are linked through (1,3)-β-linkages; adjacent β-linkages are rare or absent (Buliga et al., 1986). Approximately 10% of the polysaccharide is comprised of longer chains of adjacent (1,4)-β-linkages (Woodward et al., 1983). The DP3 and DP4 units are arranged randomly along the chain (Staudte et al., 1983). The combination of the single (1,3)-β-linkages and the random arrangement of the cellotriosyl (DP3) and cellotetraosyl (DP4) units, and hence the (1,3)-β-linkages, result in an extended polysaccharide chain that has a limited capacity to align with other (1,3;1,4)-β-glucan chains. The (1,3;1,4)-β-glucans from many cereal grains are therefore at least partly soluble in water, they adopt an asymmetrical conformation and can form gel-like structures that are believed to be functionally advantageous for non-cellulosic cell wall polysaccharides in the matrix phase of the wall (Fincher and Stone, 2004).

The ratio of the DP3:DP4 units can be used to predict the solubility of the molecule and its rheological behavior (Papageorgiou et al., 2005). High and low ratios indicate a predominance of cellotriosyl and cellotetraosyl residues, respectively, and in both cases the conformation of the polysaccharide becomes more uniform and hence more capable of aligning into insoluble aggregates (Burton et al., 2010). High and low ratios are characteristic of the insoluble (1,3;1,4)-β-glucans from lower plants such as horsetail ferns and fungi (Burton et al., 2010). The DP3:DP4 ratio in (1,3;1,4)-β-glucans from the Poaceae have intermediate values, usually around 2–3:1 (Trafford and Fincher, 2014). It would appear that (1,3;1,4)-β-glucans with these structures and physical properties have evolved and are retained by the grasses for functional reasons. Nevertheless, the ratios vary considerably across cereal species (**Table 1**; Burton and Fincher, 2012) and grains in which (1,3;1,4)-β-glucans are particularly abundant often have a lower DP3:DP4 ratio and are more soluble (Trafford and Fincher, 2014). The exception here is the relatively insoluble (1,3;1,4)-β-glucan in the grain of *B. distachyon*, where this polysaccharide has a ratio of 5.8:1 and clearly has evolved to perform a storage function (Guillon et al., 2011).

**FIGURE 5:**
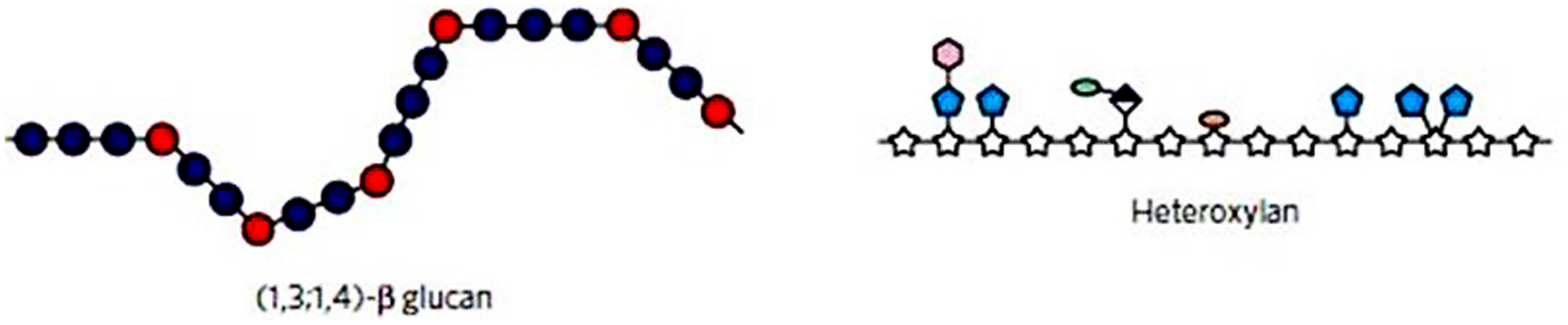
**Diagrammatical representations of the major non-cellulosic wall polysaccharides from cereal grains.** The (1,3;1,4)-β-glucan **(left)** has relatively extended regions of adjacent (1,4)-β-glucosyl residues (blue) with irregularly spaced, single (1,3)-β-glucosyl residues. The latter residues form molecular “kinks” in the polysaccharide chain and limit intermolecular alignment and microfibril formation. In the heteroxylan **(right)**, intermolecular alignment of the xylan backbone (stars) and microfibril formation is limited by steric hindrance afforded by the substituents (blue, pink, etc.). Reproduced with permission from Burton et al. (2010).

Although the chemical structures of the arabinoxylans and the (1,3;1,4)-β-glucans are quite different (**Figure 5**), their physical properties are similar and well adapted to a structural role in cell walls. This is therefore an example of convergent evolution to the extant state. Arabinoxylans are extended asymmetrical molecules by virtue of their linear (1,4)-β-xylan backbone and are partly soluble because of the steric hindrance of intermolecular aggregation afforded by their arabinofuranosyl substituents. Solubility is further influenced by acetylation and feruloylation which participate in cross-link formation between arabinoxylan and other wall components. This is exemplified in wheat endosperm walls where the degree of acetylation declines affecting solubility as the grain matures (Veličković et al., 2014) and where arabinoxylan in older walls is rendered less soluble by significant ferulate cross-linking (Saulnier et al., 2009). In contrast, the (1,3;1,4)-β-glucans are extended asymmetrical molecules by virtue of the predominance of “cellulosic” (1,4)-β-glucosyl linkages along their linear backbone and are partly soluble because of the steric hindrance of aggregation caused by the random disposition of (1,3)-β-glucosyl residues that result in randomly distributed molecular kinks in the macromolecule. Just as the solubility of arabinoxylans can be predicted from the degree of substitution and cross-linking, so too can the physical properties of (1,3;1,4)-β-glucans be predicted from their DP3:DP4 ratio. Different chemical strategies have evolved to produce the same physicochemical properties in heteroxylans and (1,3;1,4)-β-glucans.

(1,3;1,4)-β-Glucan is the predominant polysaccharide in the starchy endosperm cell walls of barley and oats and comprises about 15% of starchy endosperm cell walls in wheat grain (Mares and Stone, 1973). Recently, Veličković et al. (2014) used MALDI-MS to examine the spatial distribution of both (1,3;1,4)-β-glucan and arabinoxylan across the wheat grain. They reported higher amounts of (1,3;1,4)-β-glucan and arabinoxylan in outer endosperm regions of young grain and showed that this distribution became more even in mature grain, although cells close to the embryo had walls rich in (1,3;1,4)-β-glucan at all stages of grain development (Saulnier et al., 2009). In barley, the (1,3;1,4)-β-glucan is reported to be evenly distributed in endosperm walls by 10 DAP (Wilson et al., 2012), but little (1,3;1,4)-β-glucan was detected between 12 and 16 DAP in the peripheral starchy endosperm cells closest to the differentiating aleurone. This has also been noted in wheat (Philippe et al., 2006) but while this situation persists in wheat, in barley by 16 DAP (1,3;1,4)-β-glucan deposition has occurred in the peripheral starchy endosperm. There are clearly microdomains present across the endosperm where cell wall composition varies but the requirement for these subtle variations is presently unclear. There is also currently little information on spatial differences in the DP3:DP4 ratio of (1,3;1,4)-β-glucans across developing grain of any species, which is undoubtedly related to the lack of high resolution detection methods. However, the MALDI-MS method shows promise for these kinds of analyses. Veličković et al. (2014) were able to quantify oligosaccharides released by *in situ* digestion of (1,3;1,4)-β-glucans with lichenase and reported that the DP3:DP4 ratio was elevated to 7:1 in younger endosperm, compared with around 4:1 in mature tissue.

## EVOLUTION OF POLYSACCHARIDE SYNTHASE GENES

Many of the enzymes that catalyze the polymerization of the backbone chains of wall polysaccharides are encoded by genes that belong to the “cellulose synthase gene superfamily.” This gene family has close to 50 members in most higher plants (Richmond and Somerville, 2000; Hazen et al., 2002) and it has proved difficult to unequivocally assign functions to individual genes and some clades. The *CesA* clade encodes cellulose synthases (Pear et al., 1996; Arioli et al., 1998), but it is clear that several CesA enzymes and a number of other enzymes and/or proteins are required for an active cellulose synthesis complex (Doblin et al., 2002; Burton and Fincher, 2014). Several of the cellulose synthase-like (*Csl*) clades of the gene superfamily have been implicated in the synthesis of different wall polysaccharides. The *CslA* group of genes is likely to encode mannan and glucomannan synthases (Dhugga et al., 2004; Liepman et al., 2005). Cocuron et al. (2007) have presented evidence for a role of the *CslC* group of genes in the synthesis of the (1,4)-β-glucan backbone of xyloglucans and the genes in the *CslD* clade may be involved in cellulose synthesis, particular in cells that exhibit tip growth (Doblin et al., 2001; Favery et al., 2001; Wang et al., 2001).

A good deal of effort has been focused on the identification of genes that mediate the synthesis of the cereal grain arabinoxylans and (1,3;1,4)-β-glucans. In the case of the arabinoxylan enzymes, much of the initial work on the identification of genes involved was focused on analyses of *Arabidopsis* mutant lines and transcript profiling. These studies implicated genes from the *GT8*, *GT43*, *GT47*, and *GT61* families (Brown et al., 2007, 2009; Mitchell et al., 2007; Pena et al., 2007; Persson et al., 2007; Oikawa et al., 2010). However, these approaches are plagued with interpretative difficulties imposed by the large gene families, compensation, and pleiotropic effects in transgenic lines during proof-of-function tests, and the difficulties associated with developing reliable biochemical assays for expressed enzymes. Mitchell et al. (2007) and Pellny et al. (2012) used comparative bioinformatics analyses to predict the functions of candidate genes and concluded that genes in the GT43 and GT47 families might encode backbone (1,4)-β-xylan synthases in wheat, genes in the GT61 family might encode xylan (1,2)-α- or (1,3)-α-L-arabinosyl transferases, and that BAHD genes encode feruloyl-arabinoxylan transferases. This group recently provided additional and compelling evidence for wheat *GT61* genes, which they designated *TaXAT* for wheat, as xylan (1,3)-α-L-arabinosyl transferases (Anders et al., 2012), whilst another member of the *GT61* family in rice, called *XAX1*, was shown to be responsible for adding the xylose residues in Xylp-(1 → 2)-α-Araf-(1 → 3) substitutions (Chiniquy et al., 2012). Zeng et al. (2010) used GT43-specific antibodies to co-immunoprecipitate a complex from wheat microsomes that contained GT43, GT47, and GT75 proteins, and Lovegrove et al. (2013) used RNA interference suppression of *GT43* and *GT47* genes to reduce the total amount of arabinoxylan in wheat endosperm walls by 40–50%. Analysis of the glucuronoarabinoxylan polymer synthesized by the complex suggested a regular structure containing Xyl, Ara, and GluA in a ratio of 45:12:1. The authors suggested that this may represent a core complex in the biosynthetic process of xylans but to date we have no definitive evidence for the involvement of specific genes or proteins in the synthesis of the backbone or in the addition of certain substituents. Mortimer et al. (2010) reported that the products of two *GT8* genes mediate the addition of α-GluA and α-4-*O*-methylglucuronic acid residues to the heteroxylan of *Arabidopsis*, and Rennie et al. (2012) later established that the *GT8* gene *GUX1* performs substitution of the xylan backbone with GlcA. α-Galacturonosyl transferases that are involved in HG synthesis are also members of the *GT8* family (Yin et al., 2009). Double mutant plants for these genes (*gux1gux2*) contain xylan that is almost completely unsubstituted, but still contain wild-type amounts of the xylan backbone. This indicates that the synthesis of the backbone and its substitution can be uncoupled; a somewhat surprising observation when the behavior of such an unsubstituted and hence possibly insoluble polysaccharide in an aqueous environment is considered, although potential insolubility may be ameliorated by extensive acetylation. The domain of unknown function protein, DUF579, which was reported by Jensen et al. (2011) to be involved in xylan biosynthesis, has since been shown to encode a glucuronoxylan 4-*O*-methyl transferase that catalyzes the methyl etherification of C(O)4 of glucuronyl residues in heteroxylans of *Arabidopsis* (Urbanowicz et al., 2012).

The genes involved in the biosynthesis of (1,3;1,4)-β-glucans are reasonably well defined and include members of the *CslF* and *CslH* clades of the cellulose synthase gene superfamily. These genes are found only in the Poaceae (Hazen et al., 2002) and when transformed into *Arabidopsis thaliana* mediate the biosynthesis of (1,3;1,4)-β-glucans in the walls of transgenic plants (Burton et al., 2006; Doblin et al., 2009). As a dicotyledon, *Arabidopsis* does not normally have (1,3;1,4)-β-glucans in its walls and does not have *CslF* or *CslH* genes. These genes are members of smaller gene sub-families that contain about 10 *CslF* genes and just a few *CslH* genes (Burton and Fincher, 2012). It has not yet been demonstrated that all genes in these two clades encode (1,3;1,4)-β-glucan synthases. Additional evidence for the involvement of these genes in (1,3;1,4)-β-glucan synthesis was obtained through over-expression in barley of the *CslF6* gene driven by an endosperm-specific promoter. This resulted in increases of more than 80% in (1,3;1,4)-β-glucan content in the transgenic barley grain (Burton et al., 2010). Similarly, a mutant barley line in which there is a lesion in the *CslF6* gene has no (1,3;1,4)-β-glucan in its grain (Taketa et al., 2012). It is worth noting that the *CslF6* gene might act in concert with other proteins or enzymes during (1,3;1,4)-β-glucan synthesis and to investigate this possibility genome-wide association mapping has been used in attempts to identify other genes that might contribute to the biosynthesis or regulation of (1,3;1,4)-β-glucan synthesis (Rasmussen and Shu, 2014).

Given that the Poaceae evolved relatively recently (Feuillet et al., 2008) and that (1,3;1,4)-β-glucans are largely restricted to the Poaceae in higher plants (Harris and Fincher, 2009), it seems likely that the *CslF* and *CslH* clades evolved from other clades in the cellulose synthase gene superfamily. The *CslF* and *CslH* clades are not particularly close on the phylogenetic tree (Farrokhi et al., 2006) and this suggests that genes involved in (1,3;1,4)-β-glucan synthesis might have evolved independently on at least two occasions (Fincher, 2009). Whether these evolutionary events were based on duplication and ensuing steady changes in other *Csl* genes or whether recombination caused domain swapping in enzymes that resulted in genes encoding the (1,3;1,4)-β-glucan synthases is not known. However, it is clear that some competitive advantage must be associated with the presence of (1,3;1,4)-β-glucans in walls of the Poaceae and that selection pressure has retained the capacity of enzymes encoded by *CslF* and *CslH* genes to synthesize (1,3;1,4)-β-glucans. Detailed phylogenetic analyses indicate that the *CslF* genes shared a common ancestor with *CslD* genes and are now under a stationary selection barrier (Yin et al., 2009). A stationary selection barrier would suggest that the evolution of (1,3;1,4)-β-glucans has provided functional advantages for the Poaceae.

The recent availability of the three-dimensional structure of a bacterial cellulose synthase (Morgan et al., 2013) and a molecular model of a cellulose synthase from cotton (Sethaphong et al., 2013), provide new opportunities to link evolution at the gene level with the evolution of a new enzyme with the capacity for (1,3;1,4)-β-glucan synthesis. For example, the nascent (1,3;1,4)-β-glucan synthase enzymes might have evolved by virtue of subtle changes in the three-dimensional dispositions of active site residues or through changes in surface amino acid residues that are involved in protein–protein interactions. We are now in a position to test these possibilities.

## HAVE CELL WALL POLYSACCHARIDES EVOLVED A STORAGE FUNCTION?

**FIGURE 6:**
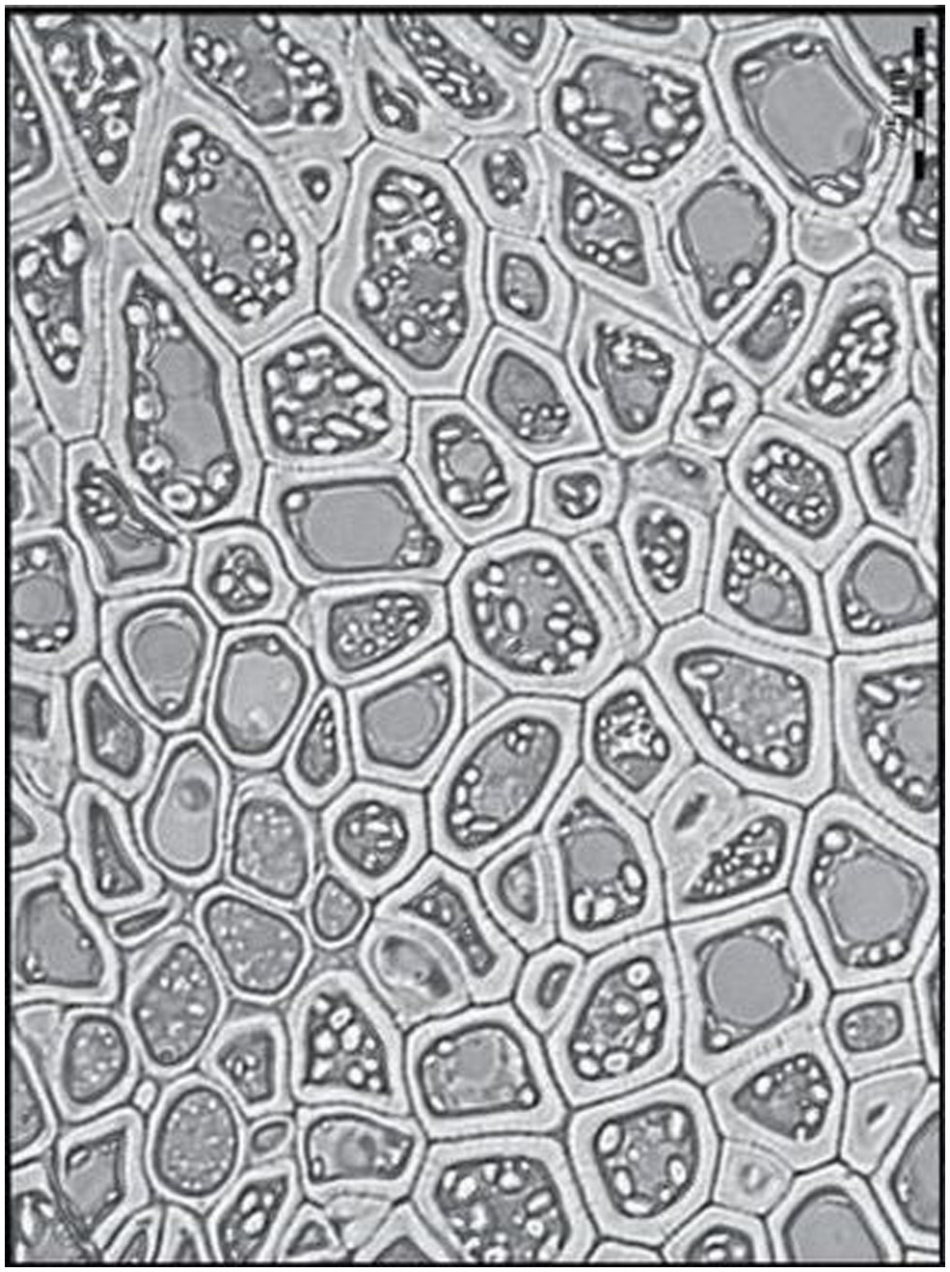
**Thick endosperm cell walls in *Brachypodium distachyon* grain.** Reproduced with permission from Trafford et al. (2013).

A striking feature of some cereal grains is the highly variable amounts of (1,3;1,4)-β-glucan that they contain; this can vary from close to zero in rice to 45% w/w in the starchy endosperm of *B. distachyon* Bd21 (Guillon et al., 2011). The starchy endosperm walls of *B. distachyon* are enormously thick compared with other cereals (**Figure 6**). In the Bd21 line there is a concomitant drop in grain starch content from values of 60–65% that are typical for grains of the Triticeae to 6% w/w (Guillon et al., 2011). Trafford et al. (2013) specifically compared grains of *B. distachyon* Bd21 and barley in terms of cell division, cell expansion, and endoreduplication during grain development. All of these processes were markedly reduced in Bd21, as were transcript levels of certain cell-cycle and starch biosynthesis genes. However, transcript levels of the (1,3;1,4)-β-glucan synthase genes, notably *BdCslF6*, were not affected. This lead to the hypothesis that the thick walls in *B. distachyon* grain are the result of continued accretion of (1,3;1,4)-β-glucan onto walls of cells that are not expanding (Trafford et al., 2013). Even though the endosperm walls of Bd21 are thicker, they contain a similar amount of (1,3;1,4)-β-glucan on a weight percentage of walls basis; the values are 80% w/w for Bd21 and about 70% w/w for barley endosperm walls. Trafford et al. (2013) suggested that if starch accumulation is a driver for cell expansion, as may occur in cereals such as wheat and barley, then the much lower level of starch synthesis in Bd21 may be primarily responsible for the reduced cell size and the concomitant re-direction of carbon into cell wall (1,3;1,4)-β-glucans.

**FIGURE 7:**
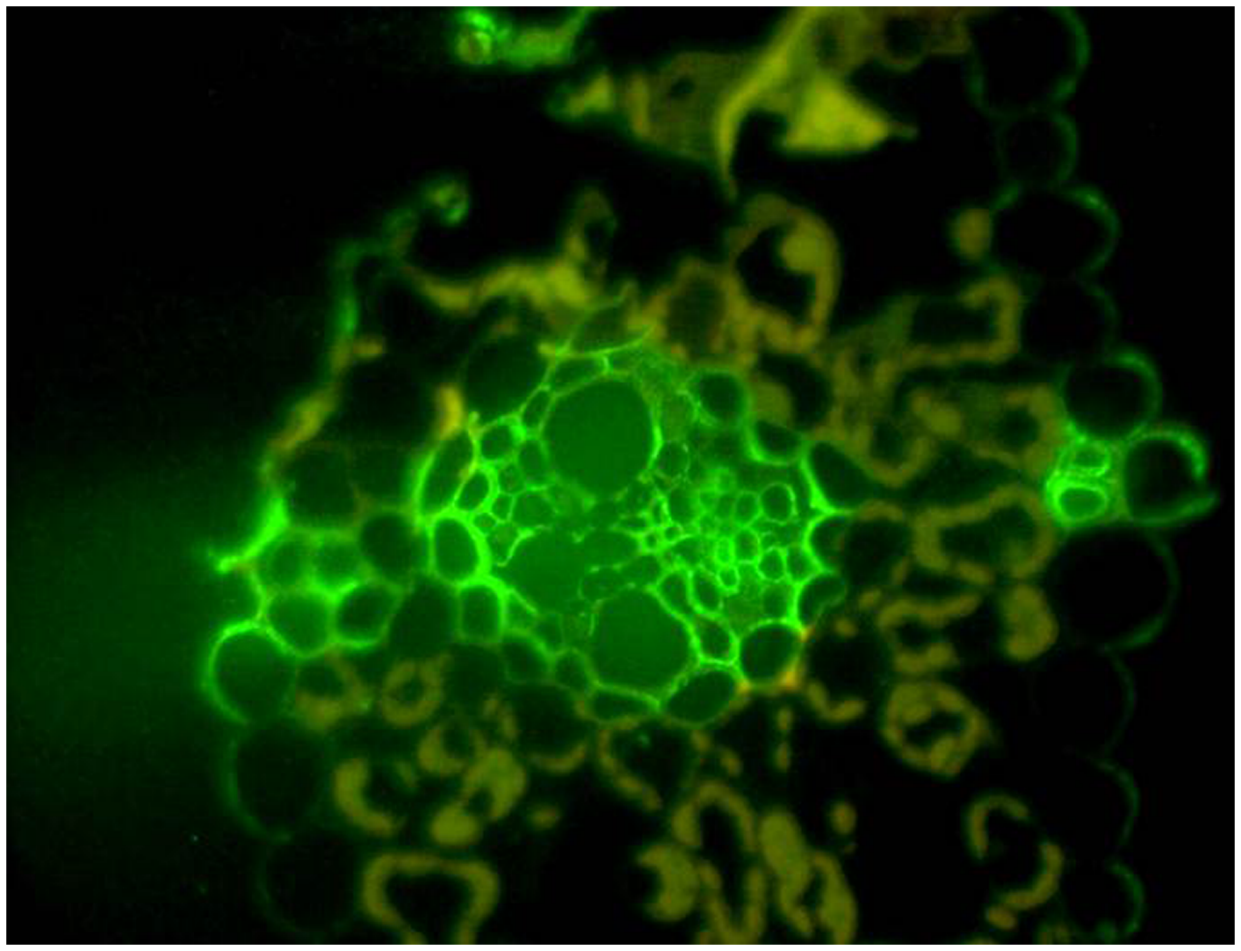
**Thin section of a young barley leaf probed with the BG1 monoclonal antibody.** The high concentration of (1,3;1,4)-β-glucans can be seen around the vasculature and the polysaccharide appears to be associated with secondary cell walls of the vasculature and other cells. Reproduced with permission from Burton et al. (2011).

The reasons for the variability of (1,3;1,4)-β-glucan content in cereal grains is not known, but it has been suggested that this polysaccharide acts as a secondary store of metabolizable glucose and that this function might be the key to the adoption of (1,3;1,4)-β-glucans during the evolution of the grasses (Burton and Fincher, 2012). It is clear that (1,3;1,4)-β-glucans are not essential structural components of cell walls in the Poaceae, because their levels are very low in some species and in many tissues of species that have high levels in their grain. It is equally clear that *B. distachyon* uses (1,3;1,4)-β-glucans as a storage polysaccharide in its grain, where small amounts of starch are present (Guillon et al., 2011). There is also indirect evidence that (1,3;1,4)-β-glucans are used as an alternative source of metabolizable energy in the leaves of barley seedlings (Roulin et al., 2002). During daylight hours, the (1,3;1,4)-β-glucan content of the leaves is about 10% w/w, but this rapidly decreases to close to zero when the plants are placed in the dark. Levels of the degradative enzymes, (1,3;1,4)-β-glucan endohydrolase isoenzyme EI and a broad-specificity β-glucan exohydrolase, increase when the plants are placed in the dark. When the lights are turned on again, the (1,3;1,4)-β-glucans rise to initial levels (Roulin et al., 2002). It has been argued that (1,3;1,4)-β-glucans would be a better short term form of stored glucose than starch, because they require a relatively simple enzyme system for both synthesis and subsequent depolymerization, and they can be deposited in the wall without the complexities of plastidial starch granule synthesis (Burton and Fincher, 2012). Might this be the reason that (1,3;1,4)-β-glucans are concentrated near the vasculature of young barley leaves (**Figure 7**)?

In the majority of cereal grains that have been examined, starch is the major storage carbohydrate. In *B. distachyon* storage metabolism seems to have shifted to (1,3;1,4)-β-glucan for unknown reasons, although disadvantageous mutations in starch synthase and cell cycle genes are possible explanations (Trafford et al., 2013). Grain anatomy, morphology, and development of *B. distachyon* has been well described (Guillon et al., 2011; Opanowicz et al., 2011; Hands and Drea, 2012; Hands et al., 2012) and grain characteristics have been compared with other domesticated cereals and non-crop species (Hands et al., 2012; Trafford et al., 2013). It is clear that the use of cell wall polysaccharides as a major source of storage carbohydrate in grains and seeds is not confined to *B. distachyon.* Of the grasses, species of *Bromus*, notably *Bromus mollis* (Hands and Drea, 2012), also possess thickened endosperm walls and a reduced starch content, suggesting that such a shift from starch to cell wall polysaccharides occurs elsewhere in the grasses but in general terms is unusual. However, a significant number of dicotyledonous seeds use cell wall polysaccharides rather than starch as the main storage medium in the endosperm (Buckeridge, 2010). These include mannans in coffee, lettuce, and tomato, glucomannan in orchids and galactomannans in legumes such as guar, fenugreek, and carob (Campbell and Reid, 1982; McCleary et al., 1985). In other examples, cell wall arabinogalactans are found as storage reserves in lupins, and xyloglucans are found in tamarind (Kumar and Bhattacharya, 2008) and nasturtium cotyledons (Edwards et al., 1985). Again, the shift from starch to wall polysaccharides is not widespread, but is seen in isolated species or genera.

Where an alternative storage carbohydrate is found in storage tissues, one would expect to see a battery of corresponding hydrolytic enzymes expressed in the germinated grain or seed, to catalyze the efficient breakdown of the polysaccharide into component sugars for use by the growing embryo and young seedling. There is little information available on germination processes in *B. distachyon* grain but it would be interesting to see if the balance of hydrolytic enzymes has also adjusted to the paucity of starch and the dominance of (1,3;1,4)-β-glucan.

## CONCLUDING REMARKS

The distinguishing features of cell walls in the grasses include the adoption of heteroxylans as the “core” non-cellulosic polysaccharide and the corresponding lower levels of xyloglucans and pectic polysaccharides. The widespread adoption of (1,3;1,4)-β-glucans in the Poaceae family also distinguishes the grasses from other monocotyledonous and dicotyledonous plants, although it is intriguing that (1,3;1,4)-β-glucans do not appear to be an essential structural component of walls in these species. One might question whether these distinguishing characteristics of cell walls of the grasses and their grain might in any way contribute to the obvious evolutionary success of the Poaceae family. It has been estimated that the grasses, which have appeared relatively recently in evolutionary history (Feuillet et al., 2008), now dominate plant ecosystems of about 20% of terrestrial land on the planet (Gaut, 2002). Does cell wall composition contribute to the ecological dominance of the grasses? Is the widespread adoption of (1,3;1,4)-β-glucans in walls of grass species and their possible function as a secondary source of metabolizable glucose important for the evolutionary success of grasses? It is clear that the (1,3;1,4)-β-glucans have appeared in other plant species, including in the walls of primitive dicots and in fungi, but they are widespread only in the Poaceae.

The non-cellulosic wall polysaccharides of plants are important components of dietary fiber and increased intake of dietary fiber has been advocated to reduce the risk of contracting serious human diseases, including colorectal cancer, type II diabetes, and cardiovascular disease (Collins et al., 2010; Burton and Fincher, 2014). Over-expression of the *HvCslF6* gene in barley led to more than 50% increases in dietary fiber in the transgenic grain (Burton et al., 2011). Given that cereal species are probably the most important source of caloric intake on Earth, one can predict that the non-cellulosic polysaccharides of cereal grains will play an important future role in human health. Conversely, the non-cellulosic wall polysaccharides present a number of technical problems in the malting and brewing industries and in animal feedstock formulations. It is therefore likely that the genetics, cell biology, and biochemistry of these polysaccharides and the enzymes that are responsible for their synthesis will remain the subjects of research interest in the immediate future. It can also be anticipated that advances in high-throughput genomics technologies, the increasing availability of complete genome sequences, and the continuing development of *in situ* methods will greatly facilitate that research.

## Conflict of Interest Statement

The authors declare that the research was conducted in the absence of any commercial or financial relationships that could be construed as a potential conflict of interest.
